# Risk factors for gastroenteritis associated with canal swimming in two cities in the Netherlands during the summer of 2015: A prospective study

**DOI:** 10.1371/journal.pone.0174732

**Published:** 2017-04-03

**Authors:** Rosa Joosten, Gerard Sonder, Saara Parkkali, Diederik Brandwagt, Ewout Fanoy, Lapo Mughini-Gras, Willemijn Lodder, Erik Ruland, Evelien Siedenburg, Suzanne Kliffen, Wilfrid van Pelt

**Affiliations:** 1 Public Health Service Utrecht Region, Zeist, the Netherlands; 2 Public Health Service Amsterdam, Amsterdam, the Netherlands; 3 Division of Infectious Diseases, Department of Internal Medicine, Academic Medical Center (AMC), University of Amsterdam, Amsterdam, the Netherlands; 4 Centre for Infectious Diseases, Epidemiology and Surveillance, National Institute for Public Health and the Environment (RIVM), Bilthoven, the Netherlands; 5 European Programme for Intervention Epidemiology Training (EPIET), European Centre for Disease Prevention and Control (ECDC), Stockholm, Sweden; Universita degli Studi di Parma, ITALY

## Abstract

Urban canal swimming events are popular in the Netherlands. In 2015, two city canal swimming events took place, in Utrecht (Utrecht Singel Swim, USS) and in Amsterdam (Amsterdam City Swim, ACS). This prospective study characterizes the health risks associated with swimming in urban waters. Online questionnaires were sent to 160 (USS) and 2,692 (ACS) participants, with relatives of participants who did not swim completing the questionnaire as a control. Swimming water specimens and stool specimens of diarrheic participants in the ACS group were analysed. A total of 49% of USS and 51% of ACS swimmers returned their questionnaires. Nine percent of USS swimmers and 4% of non-swimmers reported gastrointestinal complaints (aRR 2.1; 95% CI: 0.3–16), while a total of 31% of ACS swimmers and 5% of non-swimmers reported gastrointestinal complaints (aRR 6.3; 95% CI: 4.1–9.5). AGI risk among ACS participants was directly related to increasing number of mouthfuls of water swallowed. Various norovirus genotypes were detected in five out of seven stool specimens taken from ACS participants and in all three tested ACS water samples. We conclude that the AGI risk among open-water swimmers in urban areas depends on the circumstances around the event. The epidemiological curve, the statistical association between swimming and AGI, and the microbiological evidence for norovirus in stool and water specimens suggest that AGI outbreak after the ACS event was due to water contamination by multiple norovirus strains, which is possibly linked to sewage overflow due to prior heavy rainfall. There is need for more targeted preventive measurements and recommendations for organizers, municipal authorities and participants to prevent this reoccurring in the future.

## Introduction

In the Netherlands, swimming events in urban canal water have become increasingly popular. The health risks associated with these events are under debate but gastrointestinal outbreaks have been previously reported after exposure to untreated recreational water [1–4]. Water quality measures of recreational water are typically based on intestinal enterococci and *E*. *coli* levels as indicators of faecal contamination. However, some outbreaks were reported even when recreational water showed normal levels of intestinal enterococci and *E*. *coli* [1, 5, 6]. Additionally, high levels of intestinal enterococci and *E*. *coli* are not always an indicator of gastroenteritis outbreaks, especially those outbreaks caused by viral pathogens [6, 7]. High testing costs and long laboratory turnaround times also make it difficult for municipalities and public health services to monitor swimming water [8]. In the United States, beaches at inland lakes were subject to multiple studies [4, 9] and a significant correlation was found between the frequency of acute gastrointestinal illness (AGI) and *E*. *coli* concentrations with 3.2-fold increased odds of AGI occurring among those who had been in contact with this untreated recreational water [4]. Hand contact with floodwater has also been described as a risk factor for gastrointestinal complaints in a cross-sectional survey in the Netherlands after a period of heavy rainfall [10, 11].

Urban canal water is untreated and is not considered official swimming water. According to the European Union (EU) norm [12], official swimming water is appointed by the government, it must be monitored and management measurements must be taken in case of contamination. To state that the water quality is ‘good’, *E*.*coli* and intestinal enterococci levels should not exceed the EU norm of 1,000 CFU/dl and 400 CFU/dl, respectively [12]. Canal water can be contaminated by discharge of raw sewage from houseboats, sewage effluent, dog and bird faeces and from faecal material after heavy rainfall due to flooding. Bacterial and viral pathogens were detected in canal water during a surveillance study in Amsterdam in 2008. Detected pathogens were *Campylobacter*, *Salmonella*, *Cryptosporidium*, *Giardia* spp., rotavirus, norovirus and enterovirus [13]. Pathogen levels in urban canals are subject to frequent changes.

Outbreaks of gastrointestinal and other health complaints after swimming events in untreated recreational water have been described recently [3, 14]. As measurements of faecal indicator levels are not fully predictive for gastrointestinal outbreaks [5], more investigation is needed to gain a better understanding of the health risks associated with swimming in urban canal water. We conducted a prospective cohort study to investigate the health risks associated with swimming in urban canal water in the Netherlands in order to determine better preventive measures and recommendations for organizers, municipal authorities and participants [15].

## Methods

### Study design

Two prospective cohort studies were performed using questionnaires and laboratory investigations on two annual city swim fundraising events for Amyotrophic Lateral Sclerosis (ALS). The first event, the Utrecht Singel Swim (USS) was held on 14 June 2015, where 160 participants swam distances of 1,200 or 2,000 meters in a city canal in Utrecht. The Amsterdam City Swim (ACS) was held on 6 September 2015, where 2,692 people swam in several canals in Amsterdam for distances of 700, 1,500 or 2,000 meters.

An online questionnaire request was sent (by NetQuestionnaires) to participants 7 to 11 days after the events and participants were asked to forward the questionnaire to two or three friends or relatives who had not taken part in the swimming event so that rates of reported illness among swimmers and non-swimmers could be compared. We explained in an accompanied email that participation was voluntarily and that people could participate by clicking on the hyperlink. The questionnaire hyperlink was accessible for up to 12 days. The information collected included: age, sex, swim-related factors (i.e. distance, time swam, swimming technique, number of sips (mouthfuls of water) swallowed, previous training in open-water swimming) and detailed information about health complaints experienced after the swim and the use of catering during the event. Additional information was also collected on hypothermia related complaints, such as shivering, lethargy, slow heartbeat or breathing and sleepiness or pale skin as cold weather conditions were expected during the events. One week in advance, all participants were informed about the study by email from the event organisations regarding the purpose of the study, the voluntariness of the study, and the instruction for postponing the email to friends or relatives.

#### Case definition

We defined a gastrointestinal case for USS and ACS if diarrhoea and/or vomiting were reported up to seven days after the event. Skin complaints were defined as rash, red spots or signs of skin infection. Respiratory complaints were defined as having had a cold, coughing or dyspnoea (laboured breathing).

### Laboratory and environmental investigations

The swimming in both events took place in canal waters. In the USS event, no water specimens were collected; on the day of the ACS event three 1.5 litre water specimens were collected from three different locations along the swimming track. The Public Health Service (PHS) Amsterdam asked ACS participants to collect their stool specimens in case of diarrhoea complaints after the event and send these to the PHS in order to test for viral and bacterial agents. Information about the test and the final results were given by telephone to the participant, informed consent was obtained verbally and stool specimens were collected by the participant themselves in plastic collecting jars which could be send to the PHS by regular post. Environmental water specimens were tested for viral (real time RT-PCR for norovirus [16] and rotavirus [17], approximately 10ml of canal water was tested) and bacterial agents (*Salmonella*, *Shigella*, *Campylobacter* and *Yersinia*). Positive RT-PCR samples were further characterized by sequence analysis [18]. Water authorities measured water temperature of the swimming water, *E*. *coli* and enterococci levels were measured and reported to the PHS.

### Data analysis

We compared rates of health complaints between swimmers and non-swimmers using χ^2^ test. We performed univariate analysis to identify factors associated with AGI in swimmers and non-swimmers. A total of 22 and 31 risk factors for AGI in USS and ACS, respectively, were assessed in a single-variable analysis. Variables with a p-value ≤ 0.2 were selected for inclusion in a multivariable binomial regression model built in a stepwise fashion only among swimmers. The effect of removing variables on the other variables was monitored; removing variables that resulted in a change of other variables ≥10% were retained in the final model. The results were corrected for gender and age. Associations were expressed as adjusted relative risks (aRR) providing 95% confidence intervals (95% CI) and p-values. Statistical analysis was performed using STATA 13.

### Ethical review

The Medical Ethics Committee (MEC) of the University Medical Center Utrecht assessed the study and concluded that it was exempt from their approval; reference number: WAG/mb/16/029335. Approval from the MEC was obtained for both the questionnaire part and the stool examination part of the study. The water test sites were not private property, but open (urban) waters. No specific permission for taking water specimens was required. Participation in the questionnaire study was totally voluntary. Swimmers and non-swimmers were kindly asked by email to voluntarily participate by clicking on the questionnaire link in the email. The purpose of the study was explained in the introduction of the questionnaire. At any time a respondent could decide to not finish the questionnaire.

## Results

### Utrecht Singel Swim cohort: Cases, symptoms and risk factors

The questionnaire response in USS was 49% (79/160) among swimmers, with the addition of 27 non-swimmers. The mean age among swimmers was 38 years (19–65 years) and 25 (33%) were male. Age and gender were comparable between swimmers and non-swimmers (p-value>0.05).

Swimming did not significantly increase the risk of experiencing any symptom.

**Fig 1:**
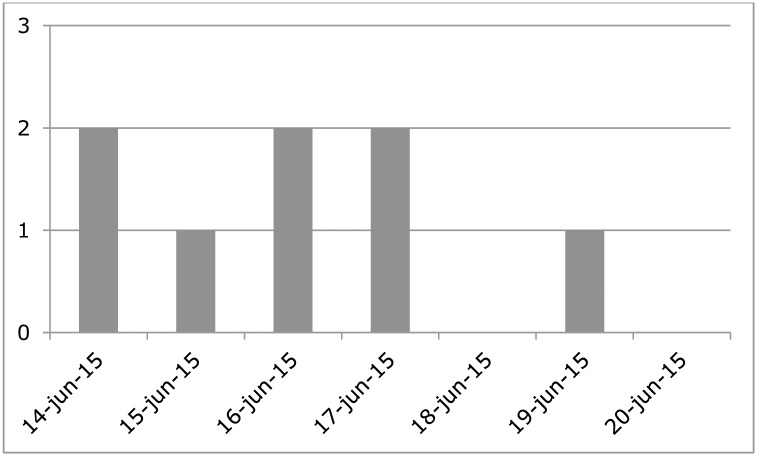
Epicurve Singel Swim Utrecht, 14 June 2015. The number of cases by date of onset of AGI following the event.

The epidemiological curve of AGI in the USS cohort showed sporadic cases but no outbreak (Fig 1). Swimmers had no significant risk for AGI compared to non-swimmers (adjusted relative risk (aRR) 2.1; 95% confidence interval (95% CI): 0.3–16.6) (Table 1). The multivariable model for AGI indicates no specific risk factors associated with illness among swimmers (Table 2). For respiratory complaints the multivariable model indicates that using medication increased the risk of respiratory complaints (aRR 6.7; 95% CI: 1.6–27.5). The medication used by the respondents was antidepressants, blood pressure or cholesterol medication, antihistamines, corticosteroid nose spray and proton pump inhibitor.

**Table 1:** Reported health complaints, risk ratios and 95% confidence intervals following the Utrecht Singel Swim and Amsterdam City Swim, 14 June 2015 and 6 September 2015.

		**Utrecht Singel Swim (USS)**						
		**Swimmers**		**Non-swimmers**			**Multivariable**	
		**(N = 79) n**	**%**	**(N = 27) n**	**%**	**(N = 106) Total**	**RR (95% CI)**^a^	**P-value**
**Health complaints** ^b^								
• Gastrointestinal tract (nausea, vomiting, diarrhoea or stomach pain)		7	9	1	4	8	2.1 (0.3–16.6)	0.495
• Respiratory tract (having a cold, coughing or dyspnoea)		4	5	0	0	4	n/a	-
• Skin (red spots, red skin, skin infection)		0	0	0	0	0	n/a	-
• Total (any health complaints)^c^		18	23	3	11	21	2.4 (0.6–9.9)	0.210
		**Amsterdam City Swim (ACS)**						
		**Swimmers**		**Non-swimmers**			**Multivariable**	
		**(N = 1,375) n**	**%**	**(N = 456) n**	**%**	**(N = 1,831) Total**	**RR (95% CI)**^a^	**P-value**
**Health complaints** ^b^								
• Gastrointestinal tract (nausea, vomiting, diarrhoea or stomach pain)		427	31	22	5	449	6.3 (4.1–9.5)	<0.001
• Respiratory tract (having a cold, coughing or dyspnoea)		171	12	17	4	188	3.3 (2.0–5.4)	<0.001
• Skin (red spots, red skin, skin infection)		24	2	0	0	24	n/a	-
• Total (any health complaints)^c^		588	43	59	13	647	3.2 (2.5–4.1)	<0.001

RR = relative risk; CI = confidence interval; n/a = not applicable

^a^ Adjusted for gender and age

^b^ Respondents were able to have separate complaints at the same time

^c^ Other complaint categories were muscle pain, cold chills, fever, red eyes, ear pain or headache

**Table 2:** Univariable and multivariable analysis on risk factors for AGI in the Singel Swim Utrecht among swimmers (*n* = 79), 14 June 2015.

			Univariable		Multivariable	
Characteristic	Total N	Cases (attack rate)	RR (95% CI)	P-value	RR (95% CI)^a^	P-value
Gender						
• Female	50	6 (12)	1	-	1	-
• Male	25	1 (4)	0.3 (0–2.6)	0.26	0.6 (0.1–4.6)	0.59
Age (years)						
• <35	34	6 (18)	1	-	*ns*	
• =>35	45	1(2)	0.1 (0–1)	0.015		
Distance swam (metres)						
• 1,200	11	1 (9)	1	-	*ns*	
• 2,000	65	6 (9)	1 (0.1–7.6)	1		
Use antacids						
• No	13	1 (8)	1	-	*ns*	
• Yes	1	1 (100)	13 (2–85.5)	0.01		
Having chronic illness						
• No	55	6 (11)	1		*ns*	
• Yes	20	0 (0)	*n/a*	0.12		
Use medication						
• No	61	4 (7)	1	-	*ns*	
• Yes ^b^	14	2 (14)	2.2 (0.4–10.7)	0.34		
Allergies						
• No	66	6 (9)	1	-	*ns*	
• Yes	9	0 (0)	*n/a*	0.35		

^a^ Adjusted for any exposure with p-value <0.2 in the univariable analysis

^b^ Medication use was antidepressants, blood pressure or cholesterol medication, antihistamines, corticosteroid nose spray and proton pump inhibitor

N: number of subjects who were exposed to the exposure category. Cases: Number with gastrointestinal complaints in week after event and being exposed to the exposure category.

*ns* = not significant *n/a* = not applicable

### Amsterdam City Swim cohort: Cases, symptoms and risk factors

The ACS questionnaire response among swimmers was 51% (1,375/2,692). Responses were available for 1,375 swimmers and 456 non-swimmers, age and gender was not reported by 63 respectively 65 swimmers, 41 non-swimmers did not report age and gender. Among swimmers, the mean age was 40 years (10–78 years) and 591 (45%) were male, whereas of the non-swimmers group the mean age was 45 years (6–78 years) and 206 (45%) were male. Swimmers were significantly younger than non-swimmers (p-value <0.001).

Among the swimmers, 427/1,375 (31%) reported gastrointestinal complaints, which was significantly more than reported by non-swimmers (22/456; 5%) (Table 1). Among swimmers 330 met the AGI case definition. Swimmers also reported significantly more respiratory tract infections (171/1,375; 12%) than non-swimmers (12/456; 4%) (Table 1).

Among all of the ACS participants with diarrhoea, 244 of 311 (79%) had recovered by the time they completed the questionnaire. The mean duration of their diarrhoeal complaints was 2.4 days (0.5–13 days). Among ACS respondents with gastrointestinal complaints, swimming was associated with disease (aRR 6.3; 95% CI: 4.1–9.5).

Hypothermia complaints appeared in 6% (86/1,375) of swimmers and nine participants were taken out of the water because of hypothermia complaints. No relation with any of the health complaints was found.

**Fig 2:**
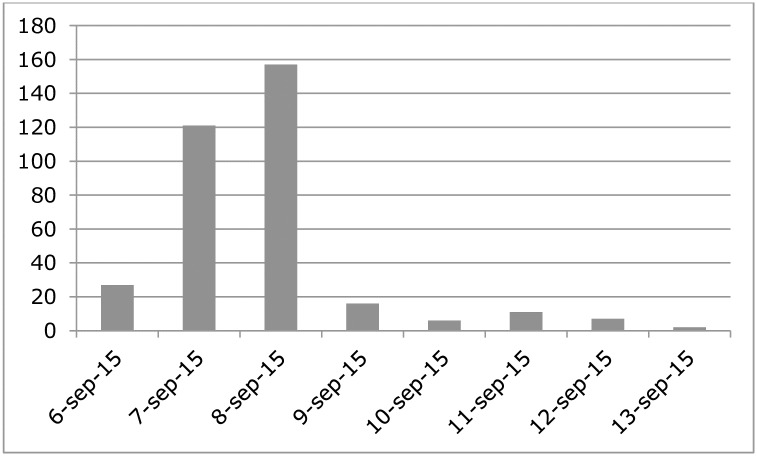
Epicurve Amsterdam City Swim, 6 September 2015. The number of cases by date of onset of AGI following the event.

The epidemiological curve of AGI in the ACS cohort suggests a point source in time, as a peak of reported AGI was observed the second and third day after the event, which was followed by a steady decline in newly reported cases (Fig 2).

Gender and age were not associated with AGI (Table 3). The median number of sips swallowed by all swimmers was 3.1 (95% CI: 3–3.2). The aRR increased per interval of sips ingested (i.e. none, one to three, more than three). Swimming duration of more than 60 minutes was borderline significant associated with AGI (aRR 1.3; 95% CI: 1–1.7). Other swim-related factors were not associated with AGI.

**Table 3:** Univariable and multivariable analysis on risk factors for AGI in the Amsterdam City Swim among swimmers (*n* = 1375), 6 September 2015.

			Univariable		Multivariable	
Characteristic	Total N	Cases (attack rate)	RR (95% CI)	P-value	RR (95% CI)^a^	P-value
Gender						
• Female	719	197 (27)	1	-	1	-
• Male	591	125 (21)	0.8 (0.6–0.9)	0.009	0.9 (0.7–1.1)	0.33
Age (years)						
• <= 14	32	7 (22)	1	-	1	-
• 15–35	399	138 (35)	1.6 (0.8–3.1)	0.14	1.3 (0.6–2.5)	0.46
• 36–45	430	95 (22)	1 (0.5–2)	0.98	0.9 (0.4–1.7)	0.68
• =>46	451	83 (18)	0.8 (0.4–1.7)	0.63	0.4 (0.4–1.4)	0.36
Distance swam (metres)						
• 700	113	17 (15)	1	-	*ns*	
• 1,500	145	35 (24)	1 (0.7–1.4)	0.97		
• 2,000	1,094	278 (25)	1.4 (1.1–1.8)	0.016		
Swimming technique						
• Backstroke	46	17 (37)	1.6 (1.1–2.3)	0.04	*ns*	
• Breaststroke	1,088	275 (25)	1.2 (0.9–1.6)	0.13		
• Freestyle	517	132 (26)	1.1 (0.9–1.3)	0.45		
Frequency of training in open water in last 3 months						
• =< 2 times	451	117 (26)	1	-	*ns*	
• >= 3 times	924	213 (23)	0.9 (0.7–1.1)	0.24		
Swallowing water						
• No sips	301	31 (10)	1	-	1	-
• 3 or less sips	642	170 (27)	2.6 (1.8–3.7)	<0.001	2.4 (1.7–3.4)	<0.001
• More than 3 sips	404	129 (32)	3.1 (2.2–4.5)	<0.001	2.9 (2–4.2)	<0.001
Minutes swam						
• =< 45	363	72 (20)	1	-	1	-
• 46–60	580	139 (24)	1 (0.8–1.2)	0.98	1 (0.8–1.3)	0.81
• =>61	404	118 (29)	1.3 (1.1–1.6)	0.004	1.3 (1–1.7)	0.06
Swam in open water in previous week						
• No	848	221 (26)	1	-	*ns*	
• Yes	500	109 (22)	0.8 (0.7–1)	0.08		
Use medication						
• No	1,135	276 (24)	1	-	*ns*	
• Yes	191	51 (27)	1.1 (0.9–1.4)	0.48		
Use antacids						
• No	161	42 (26)	1	-	*ns*	
• Yes	30	9 (30)	1.2 (0.6–2.1)	0.66		
Catering						
• No	577	153 (327)	1	-	1	-
• Yes	798	177 (22)	0.8 (0.7–1)	0.06	0.8 (0.6–0.9)	0.003

^a^ Adjusted for any exposure with p-value <0.2 in the univariable analysis

N: number of subjects who were exposed to the exposure category. Cases: Number with gastrointestinal complaints in week after event and being exposed to the exposure category.

*ns* = not significant

Chronic skin disease was a risk factor for skin complaints (aRR 17.6; 95% CI: 9.2–33.5), as was cardiovascular disease for skin complaints (aRR 19; 95% CI: 1.8–189) in the ACS. Being male showed a lower aRR for skin complaints (aRR 0.3; 95% CI: 0.1–0.9). Hypothermia did not show a significant increased risk for developing AGI (aRR 1.3; 95% CI: 1–1.8).

### Laboratory and environmental investigations

No stool specimens were collected in the USS. One week before the USS event, in the context of regular measurements performed by water authorities, environmental water specimens were tested for *E*. *coli* and intestinal enterococci levels. The EU norm of 1,000 CFU/dl and 400 CFU/dl [12] were both not exceeded in the week before the event. On the day of USS, the water temperature was 20°C [19].

Seven ACS participants with complaints of diarrhoea provided a stool specimen. Five stool specimens tested positive for norovirus (NoV) genogroup 1 (G.1), of which four specimens were a heterogeneous mixture of NoV G.1 subtypes. One of three water specimens tested positive for NoV G.1, showing different subtyping compared to the stool subtypes. Two other water specimens were found positive for NoV genogroup 2 (G.2).

Three of four patients with positive NoV stool specimens visited their GP and one patient with a negative NoV test visited their GP. Patients with positive NoV stool specimens reported several complaints; all reported diarrhoea, nausea, vomiting and stomach pain.

Two days before the ACS, on 4 September 2015, an unusually heavy rainfall in Amsterdam led to severe flooding causing sewage overflow into the city canals. As an emergency measure, in cooperation with the Regional Water Board, excessive rain water of unknown quality from non-urban locations was diverted into the canals, and canal water was pumped out of the canals to flush and clean the canal water. On the morning of the ACS event, *E*. *coli* and intestinal enterococci levels were found to be below EU norm levels. The first swimmer group started at 12.00 p.m. and at 2.24 p.m. on the day of the event, the *E*. *coli* levels had increased to 4,000–10,000 CFU/dl at four different locations along the swimming track, intestinal enterococci levels increased to a maximum of 100 CFU/dl. The water temperature of the Amsterdam canal on the day of the event was 16.5°C [20].

## Discussion

This is the first study that has examined the correlation of AGI (among other health complaints) within the context of organised open-water swimming events in urban areas. Based on the characteristic epidemiological curve, the statistical association between swimming and AGI and the microbiological findings in diarrhoeal stool and water specimens, it can be concluded that the AGI outbreak after the ACS event was probably caused by norovirus. This conclusion is furtherly supported by the trend we found between the number of mouthfuls of water swallowed and the risk to develop AGI. This finding is consistent with other studies where ingestion of water while swimming was associated with gastrointestinal complaints [2, 5, 7, 14].

This study uniquely focused on the exposure to urban water and combined questionnaire data with laboratory diagnostics. Previous outbreak studies have largely focused on the effects of swimming on AGI in official recreational water [1, 5], inland beaches [4, 7, 8], lakes [3] or marine water [14, 15]. This study has increased our understanding of the health risks of swimming in water in urban areas.

There was no increase in the reported incidence of both gastrointestinal complaints or influenza-like complaints, as reported by National Syndromic Surveillance (Nivel registrations) in the Netherlands in the week before and after both events; both syndromes were well below epidemic thresholds [21]. Therefore, and because of the difference we found between the swimmers and non-swimmers in the ACS, the increased incidences were most likely caused by swimming.

For the increased risk of respiratory symptoms among swimmers who use medication and the increased risk of skin complaints in swimmers with cardiovascular disease, we did not find an explanation. Further studies, including to the presence of respiratory pathogens in water, are needed to confirm the relation and find a possible cause.

Our findings suggest that advising participants to avoid ingesting water while swimming may mitigate the health risks related to swimming in urban water. Moreover, the use of statins or diuretics [22], younger age and female swimmers [7, 14, 23] have been described as risk factors for gastrointestinal complaints caused by norovirus. For gastroenteritis caused by bacterial pathogens, such as cholera, *Salmonella* and *Campylobacter*, the association with the use of gastric antacids is well known [24]. However, this relationship has not been reported for viral pathogens and was not found to be significant in our study. The epidemiological curve suggests a ‘point source outbreak’, the incubation time of one to two days, and the reported duration of complaints, is also compatible with the hypothesis of a viral cause.

Norovirus genotype 1 (G.1) and 2 (G.2) are the most common genotypes causing outbreaks of person-to-person transmission. Genotype 2.4 is known to be responsible for >85% of norovirus outbreaks [25, 26]. In this study, norovirus G.1 was detected in five of the seven stool specimens, which is an unusually high proportion. Genotype 1 is known for its presence in the environment in water and soil compared to norovirus G.2. Norovirus G.1 causes outbreaks in humans less frequently [26]. Norovirus was found in water specimens taken from canal water in Amsterdam in a former study [13]. A study in Norway detected norovirus G.1 and G.2 in 62% and 46% of water specimens taken from the surface water of a river, respectively, norovirus G.2 was found more often in waste water [26]. The study indicated a year-round presence of norovirus in Norwegian surface water. Information about the prevalence of pathogens in open water (i.e. rivers) would have been useful for our study.

In order to underpin a causal relation of detected pathogens among swimmers, it would have been better to collect faecal specimens among swimmers *without* complaints as well. Unfortunately, for the current study, this was not feasible because of limited resources.

At both events, *E*. *coli* and enterococci levels did not exceed EU thresholds in the days prior to the event. Previous studies state that measurements of bacterial indicators are not per se related to the presence of viral pathogens in the water [1, 5, 6, 27]. In ACS, *E*. *coli* levels exceeded the EU norm in the afternoon on the day of the event, but were at acceptable levels in the morning. To our knowledge, there is no fast diagnostic tool available to test for the presence of viral pathogens in water hours before an event. Even if such a test was available, it might be in practice impossible to cancel a large event at such short notice.

Besides bacterial coliform contamination, detection of coliphages, associated with fecal contamination, can be an indicator of the presence of human viral pathogens [28]. Certain coliphages can be very specific for human fecal bacteria. But the presence of indicator coliphages, bacteria or even viral pathogens in water does not necessarily mean that there is an increased risk for an outbreak of gastrointestinal complaints [1], depending on factors such as virus concentration in water, virus pathogenicity, swimmer’s health status and swimming behavior.

The heavy rainfall event prior to the ACS event could explain the high number of swimmers with AGI symptoms after participation, which is probably linked to norovirus contamination of the water following heavy rainfall and overflow of the sewage system in the Amstelland polder area two days before the swimming event. The different norovirus types found in stool and water specimens could reflect a multi-strain contamination of the water. Heavy rainfall events tend to increase the risk of waterborne disease outbreaks, as described in a study where 51% of waterborne disease outbreaks were preceded by precipitation events of extreme rainfall above the 90th percentile in a two-month period [8, 29]. Extreme rainfall events (i.e. more than 2 inches a day) may become more frequent in the future due to climate change [8, 11].

A water quality forecast model was developed and implemented as an early-warning system after an outbreak of AGI among participants in a marine water event in Denmark [14]. These water quality forecasts models combine the knowledge of the originating sources of the urban canal water, the sewage outflow sites and the weather forecasts, in order to optimize assessment of exposure to infectious water-borne pathogens [30]. When using these models, decisions could be made about postponing open-water swimming events depending on the water quality forecast model.

This study has some limitations. Participants were asked to send a hyperlink to the questionnaire to their relatives and ask them to complete and return the questionnaires. These might have been non-swimmers who were not present at the event, resulting in different exposure between swimmers and non-swimmers. Our attempt was to include a larger number of non-swimmers in this study in order to have a sufficient comparison group to compare swimmers and non-swimmers. To allow swimmers to forward the questionnaire to non-swimmers may not be the most ideal way to recruit non-swimmers, but this was the most convenient option here. For future events, we advise to forward the questionnaire to more than 3 relatives (5 or 6) in order to increase the number of responding non-swimmers. Furthermore, participants with existing health complaints might have been more motivated to participate in the study. Therefore, the number of AGI cases might have been overestimated. Another acknowledged limitation in our study is the time of follow up which might not be sufficient to determine whether infections with incubation periods for sometimes longer than two weeks, i.e. leptospirosis, occurred after participation. We did not test parasitic pathogens in environmental and faecal specimens.

The most likely pathogen that caused the outbreak of AGI among ACS swimmers was norovirus and water was the most likely transmission route, however we still cannot determine the definitive source of this outbreak. Two main sources are possible, the presence of norovirus in the water prior to the event (due to direct sewage overflow, contaminated polder water or boat sewage dumping) or, less likely considering the different strains detected among cases, the introduction of norovirus strains by a participant (i.e. contamination of water by illness of participating individuals or contamination by hands from contaminated surfaces of toilets, showers or railings).

## Conclusion

This study provides evidence that exposure to urban canal water while swimming under specific circumstances can increase the risk of AGI. There was a relation between the ingestion of water and the occurrence of AGI. Water may have been pre-infected with viral pathogens or contaminated by the participants. Ideally, predictive models that include weather forecasts, location-specific water flow information and sewer overflow sites would be helpful in order to assess the risk of AGI before a swimming event takes place. As we can expect more heavy rainfall events to occur due to the effects of climate change, more research on swimming events in urban water will help us to understand the risk for the occurrence of outbreaks and to clarify the risk factors and the participants’ susceptibility to gastrointestinal, respiratory or skin complaints.

## Supporting information

S1 File Questionnaire Singel Swim Utrecht 2015. (PDF)

S2 File Questionnaire Amsterdam City Swim 2015. (PDF)

S3 File Dataset. Compromised dataset of respondents Singel Swim Utrecht 2015 and Amsterdam City Swim 2015
(XLSX)

S1 Text Copy of permission for publication striking image. (PDF)
