# Supplementary material for: Risk factors for gastroenteritis associated with canal swimming in two cities in the Netherlands during the summer of 2015: A prospective study
Source: PLoS One. 2017 Apr 3;12(4):e0174732. doi: 10.1371/journal.pone.0174732 (PMC5378355; doi:10.1371/journal.pone.0174732)
Supplement: S1 File — (PDF) [file pone.0174732.s001.pdf]

Public Health Service - Study Singel Swim Utrecht – questionnaire June 2015

The PHS region Utrecht investigates if participants of the Singel Swim Utrecht have run risk on health complaints by swimming in Utrecht canals.

We hope to get more insight in the health risks of swimming in open water. Therefore a questionnaire is developed. Complaints mainly develop in between two weeks after exposure. The questions therefore relate to the period of 14-28 June. Also when you did not have had complaints, it is important to fill in this questionnaire. The purpose is to fill in the questionnaire before 6th July.

To all participants we ask to find 2 persons in their own surroundings who would also fill in the questionnaire. The reason to ask this is to compare swimmers with non-swimmers.

All information you give will be handled in privacy and is included in the secrecy of all PHS attendees. We will only communicate about the general results, not about individual persons.

Filling in the questionnaire will take 5 minutes of your time.

1. Did you participate in Singel Swim?
2. What distance did you swim? (1200, 2000 meters)
3. What is your postal code?
4. What is your date of birth?
5. What is your gender?
6. Have you had one of the complaints mentioned here in the period from Sunday 14th June until Sunday 28th June? (more options can be filled in)
    - Nausea
    - Vomiting
    - Headache
    - Fever (>38 degrees)
    - Cold shivering
    - Stomach pain
    - Diarrhea
    - Muscle pain or arthralgia
    - Red eyes
    - Ear pain
    - Having a cold, coughing or dyspnea
    - Red spots on the skin
    - Other complaints, namely:….
7. When did this complaint start?
8. When was this complaint over? (data options or still complaints)
9. Have you been to a general practitioner because of your complaints?
10. Did the GP send in materials for laboratory investigation? (for example stool, blood, urine, nose- or throat glue, wound liquid)
11. What kind of material was send for investigation?
12. What were the results of this test?
13. Do you give permission to contact the GP in case we want to ask additional questions? (fill in the information about the GP)
14. Did other persons in your surrounding report any comparable complaints in the week before you started having complaints? (No, family, friends/neighbors, others….)
15. Did other persons in your surrounding report any comparable complaints in the week after you started having complaints? (No, family, friends/neighbors, others….)

16. What do you think could have been the cause of your complaint?
17. In the previous year, have you had any of these chronic diseases? (more options can be filled in)
    - No, none
    - Absence of the spleen
    - Diabetes
    - Rheuma/ rheumatic arthritis
    - Liver disease, namely:
    - Kidney disease, namely:
    - Cardiovascular disease
    - Leukemia or other cancer type, namely:
    - Immunodeficiency, namely:
    - Lung disease, namely:
    - Disease of the gastrointestinal tract, namely:
    - Hay fever or other allergy, namely:
    - Skin disease or open wounds, namely:
    - Transplantation, namely:
    - Received in last 3 months immunoglobulins, namely:
    - Received in last 3 months blood transfusion, namely:
    - Another (severe) disease, namely:
18. Do you use any medication?
19. Do you use medication of which you know reduces your immunity? Namely:
20. Do you use antacids? Namely:
21. Do you give permission to the PHS to contact you when we have additional questions?
22. If yes, what is your name and surname?
23. What is your telephone number and email address?
