# Supplementary material for: Risk factors for gastroenteritis associated with canal swimming in two cities in the Netherlands during the summer of 2015: A prospective study
Source: PLoS One. 2017 Apr 3;12(4):e0174732. doi: 10.1371/journal.pone.0174732 (PMC5378355; doi:10.1371/journal.pone.0174732)
Supplement: S2 File — (PDF) [file pone.0174732.s002.pdf]

Public Health Service - Study Amsterdam City Swim – questionnaire September 2015

Thank you for participation!
Please fill in the questionnaire before 26th September.
This questionnaire is filled in by participants and non-participants of the Amsterdam City Swim.
The questions relate to the period of Sunday 6th September until Sunday 20th September.
Filling in will take 5 till 10 minutes from your time. The questionnaire includes 108 questions.

1. Did you participate in the Amsterdam City Swim?
2. What distance did you swim? (700, 1500, 2000)
3. How many minutes did you swim? (…)
4. Do you have a good condition? (for example do you sport more than a few times a week)
5. Where you trained to swim 700/1500/2000 meters in the open air?
6. How often did you, in the 3 months before the event, swim 700/1500/2000 meters in a stretch?
7. Did you wear a wetsuit during swimming?
8. What kind of wetsuit did you wear? (shorty, long john, full wetsuit, other, namely…)
9. How did your wetsuit connect to your body? (good, to wide, to tight)
10. Which technique did you use while swimming? (breaststroke, freestyle, other, namely….)
11. Did you, accidentally, ingest water while swimming?
12. How many sips you treasure having swallowed? (0, 1, 2, 3, 4-5, 6-9, 10 or more)
13. Did you swim in open water in the week previous to the event?
14. Have you had one of the complaints mentioned here in the period from 6th September until now? (more options can be filled in)
    - Nausea
    - Vomiting
    - Headache
    - Fever (>38 degrees)
    - Cold shivering
    - Stomach pain
    - Diarrhea
    - Muscle pain or arthralgia
    - Red eyes
    - Ear pain
    - Having a cold, coughing or dyspnea
    - Red spots on the skin
    - Symptoms of hypothermia (i.e. shivering, lethargy, drowsiness, pale skin, sleepiness, slow pulse and breathing)
    - Other complaints, namely:….
15. When did this complaint start?
16. Do you still have this complaint?
17. When was this complaint over?
18. In case of diarrhea: how often in 24 hours did you had complaints of diarrhea?
19. In case of hypothermia: did you had this before? Were you taken out of the water due to hypothermia?
20. Have you been to a general practitioner because of your complaints?
21. Did the GP send in materials for laboratory investigation? (for example stool, blood, urine, nose- or throat glue, wound liquid)
22. What kind of material was send for investigation?
23. What were the results of this test?

24. Do you give permission to contact the GP in case we want to ask additional questions? (fill in the information about the GP)
25. What do you think could have been the cause of your complaint?
26. Did other persons in your surrounding report any comparable complaints in the week before you started having complaints? (No, family, friends/neighbors, others….)
27. Did other persons in your surrounding report any comparable complaints in the week after you started having complaints? (No, family, friends/neighbors, others….)
28. Have you been abroad in the week previous to the event?
29. Which country have you been visiting?
30. Do you have any of these chronic diseases? (more options can be filled in)
    - No, none
    - Absence of the spleen
    - Diabetes
    - Rheuma/ rheumatic arthritis
    - Liver disease, namely:
    - Kidney disease, namely:
    - Cardiovascular disease
    - Leukemia or other cancer type, namely:
    - Immunodeficiency, namely:
    - Lung disease, namely:
    - Disease of the gastrointestinal tract, namely:
    - Hay fever or other allergy, namely:
    - Skin disease or open wounds, namely:
    - Transplantation, namely:
    - Received in last 3 months immunoglobulins, namely:
    - Received in last 3 months blood transfusion, namely:
    - Another (severe) disease, namely:
31. Do you use any medication?
32. Do you use medication of which you know reduces your immunity? Namely:
33. Do you use antacids? Namely:
34. What is your age?
35. What is your gender?
36. What is your length (in cm)?
37. What is your weight (in kg)?
38. What are the first numbers of your postal code?
39. Did you use the catering service on the day of the event?
40. Do you give permission to the PHS to contact you when we have additional questions?
41. If yes, what is your name and surname?
42. What is telephone number and email address?
