# Supplementary material for: Risk factors for gastroenteritis associated with canal swimming in two cities in the Netherlands during the summer of 2015: A prospective study
Source: PLoS One. 2017 Apr 3;12(4):e0174732. doi: 10.1371/journal.pone.0174732 (PMC5378355; doi:10.1371/journal.pone.0174732)
Supplement: S1 Text — (PDF) [file pone.0174732.s004.pdf]

Van: coco@nationalebeeldbank.nl [mailto:coco@nationalebeeldbank.nl] **Namens** Support | Nationale Beeldbank
Verzonden: maandag 16 januari 2017 11:26
Aan: Rosa Joosten
Onderwerp: Re: Contact verzoek: Rosa Joosten

Dear Rosa,

This is to certify that you may use this editorial without any restrictions. We make no separate attachments here.

Sincerely,

Coco Broeken
Department Support

Nationale Beeldbank is onderdeel van Het Beeldhuis van Nederland
Beeldstudio | Richhistory | Visual Impact | Nature in Stock | Stichting Polyvisie

Algemeen: +31 (0)88 - 98 98 700
Fotografen:+31 (0)88 - 98 98 703

Willebrordusstraat 10
3037 TR Rotterdam

www.nationalebeeldbank.nl
